# Rev. George W. Taylor, M. D.

**Published:** 1913-03

**Authors:** 


Rev. George W. Taylor, M. D., died at his home in Princeton, Ill., January 26, aged 97, of senile debility. He graduated in medicine in 1853, from the Syracuse Medical College, an eclectic institution, organized in 1850 and extinct since 1857. Dr. Taylor retired from medical practice many years ago and became a minister of the Christian Church.

